# Parasitic versus nutritional regulation of natural fish populations

**DOI:** 10.1002/ece3.4391

**Published:** 2018-08-05

**Authors:** Amélie Frantz, Marie‐Elodie Perga, Jean Guillard

**Affiliations:** ^1^ UMR 042 CARRTEL INRA – University Savoie Mont Blanc Thonon‐les‐Bains France; ^2^ Institute of Earth Surface Dynamics University of Lausanne Lausanne Switzerland

**Keywords:** allometric growth, fatty acid composition, food quality, lake phosphorus concentration, parasitism, *Perca fluviatilis*, stable isotopes, *Triaenophorus nodulosus*

## Abstract

Although parasites are expected to affect their host's fitness, quantitative proof for impacts of parasitism on wild populations is hampered by confounding environmental factors, including dietary resource. Herein, we evaluate whether the physiological conditions of European perch (*Perca fluviatilis*) in three large peri‐alpine lakes (Geneva, Annecy, and Bourget) depend on (a) the nutritional status of the juvenile fish, as revealed by stable isotope and fatty acid compositions, (b) the prevalence of the tapeworm *Triaenophorus nodulosus*, a parasite transmitted to perch through copepod preys, or (c) interactive effects of both factors. At the scale of lake populations, the deficit in growth and fat storage of juvenile perch during their first summer coincides with a high parasite prevalence and also a low quality of dietary resource. Yet, at the individual level, parasites had no evident effect on the growth of the juvenile perch, while impacts on fat storage appeared only at the highest prevalence of the most infected lake. Fatty acid and stable isotope analyses of fish tissue do not reveal any impact of *T. nodulosus* on diet, physiology, and feeding behaviour of fish within lakes. Overall, we found a low impact of parasitism on the physiological condition and trophic status of juvenile perch at the end of their first summer. We find instead that juvenile perch growth and fat storage, both factors tied to their winter survival, are under strong nutritional constraints. However, the coinciding nutritional constraints and parasite prevalence of perch juveniles in these three lakes may result from the indirect effect of lake nutrient concentrations, which, as a major control of zooplankton communities, simultaneously regulate both the dietary quality of fish prey and the host–parasite encounter rates.

## 1 INTRODUCTION

Parasites, by definition, impose a cost on their hosts’ resources, often affecting their life history traits (Combes, 2001). Impacts of parasitism at the individual host level are expected to cascade onto the host population dynamics, which, under the simplest scenario, will result in a decrease in the host population density. Despite a number of theoretical predictions and outputs from controlled experiments (Anderson & May, 1978; May & Anderson, 1978), empirical evidence that parasites can actually regulate wild populations and cycles in nature is scarce (but see Watson, 2013). Indeed, assessing the effective parasite check on natural populations is challenging as parasite impacts cannot be inferred from incidence rates alone and comparative evidence from infected and noninfected populations is difficult to obtain (Washburn, Mercer, & Anderson, 1991). Quantitative proof for impacts of parasitism on wild populations may also be hampered by the fact that most natural host populations are infected by multiple parasites species coupled with other confounding environmental factors (abiotic conditions, nutritional resources, competition, predation) which influence the host population as well (Decaestecker, Declerck, De Meester, & Ebert, 2005). However, a recent meta‐analysis of 38 experimental studies on the cost of parasites from individual — to population—level measures of natural, free‐ranging hosts revealed an overall moderate but negative effect of parasites (Watson, 2013). Thus, the hypothesis that parasitism is a regulatory force on wild vertebrate host populations should be considered in population dynamics models (Lafferty, Dobson, & Kuris, 2006).

The next step is to test for and quantify the effective contribution of parasitism in natural population control and compare to trophic regulators, which are generally considered the drivers of the structure and dynamics of natural populations (Paine, 1980). Within this broader picture, the role of parasites may be more subtle than purely additive to the other drivers. Nutrition is a key environmental factor shaping immune defences and host susceptibility to infection (Scrimshaw, 1959 *in* Cornet, Bichet, Larcombe, Faivre, & Sorci, 2014). Changes in host nutritional status, in terms of both quantity and quality, can have profound repercussions on the dynamics of infectious diseases and moderate the consequences of the parasite on the host population dynamics (Aalto, Decaestecker, & Pulkkinen, 2015; Lange, Reuter, Ebert, Muylaert, & Decaestecker, 2014; Washburn et al., 1991). For instance, the regulatory role of the ciliate *Lambornella clarki* on the larval population of its mosquito host *Aedes sierrensis* has been shown, both in the laboratory and in the field, to vary with the availability of food resources (Washburn et al., 1991). When the host develops with sufficient food, the parasite effect is additive, increasing mortality and reducing populations. When hosts are food limited, however, effects shift to compensatory (no effect on host abundance) or depensatory (higher abundance of the hosts; Washburn et al., 1991). Along with food quantity, food quality might also affect the outcome of epidemics at the population level by its contradictory effects on both the host and parasite population dynamics (Aalto et al., 2015; Pulkkinen, Wojewodzic, & Hessen, 2014). For instance, high‐quality food promotes the growth of *Daphnia dentifera* which, when infected by the fungus *Metschnikowia bicuspidata*, produces more spores than hosts fed at lower food quality (Duffy et al., 2008). However, as infected hosts produce fewer offspring than uninfected individuals, the infection prevalence declines with the resource quality (Duffy et al., 2008). Besides, a better nutritional status of the host does not necessarily limit the parasite impact at individual — and population—scales. In a bird–malaria system, parasites more successfully controlled nonsupplemented birds, but hosts with a better nutritional status paid a higher cost of infection (Cornet et al., 2014). Therefore, interactions between epidemics and dietary components may result in contradictory population‐level outcomes depending on the host–parasite model and environmental context (Aalto et al., 2015; Pulkkinen et al., 2014). The relative and potentially interacting effects of resource and parasitic controls on natural population dynamics need to be further investigated in the field.

To address this question, we focused on the life history traits of juvenile European perch (*Perca fluviatilis*) in three large peri‐alpine lakes (Geneva, Annecy, and Bourget). The lakes are very similar in their history of fish populations, but vary in terms of both nutritional status and the prevalence of the tapeworm *Triaenophorus nodulosus*. In Lake Annecy, most of the young‐of‐the‐year (YOY) perch die off during late autumn and consequently captures of 1 +  fish are rare (Guillard, Perga, Colon, & Angeli, 2006). Perch recruitment, that is, fish surviving their first year, in the two other lakes is much higher (Hofmann & Raymond, 2014; Jacquet et al., 2015).

The lower winter survival for Lake Annecy YOY perch might result from two potentially interacting causes. The first hypothesis is nutritional limitation. All three lakes are currently under re‐oligotrophication after a phase of human nutrient enrichment (i.e., eutrophication) in the mid‐twentieth century (Gerdeaux, Anneville, & Hefti, 2006). Nutrient abatement measures were, however, undertaken earlier in Lake Annecy than in lakes Bourget and Geneva. As a result, Lake Annecy is now oligotrophic, that is, phosphate concentration in the water is <4 μg/L, while the other two lakes are mesotrophic (phosphate concentration 15–20 μg/L), data from OLA, Observatory of LAkes (http://www6.inra.fr/soere-ola, recorded in early fall 2015, ©SOERE OLA‐IS, AnaEE‐France, INRA Thonon‐les‐Bains, CISALB, CIPEL, SILA, developed by Eco‐Informatics ORE INRA Team). In these three phosphorus‐limited ecosystems, the phosphate concentration in the water strongly determines plankton quantity and quality (Müller‐Navarra & Lampert, 1996) and YOY perch are zooplanktivorous (Masson, Angeli, Guillard, & Pinel‐Alloul, 2001). The dietary hypothesis posits that the lower availability and nutritional quality of the food resource for YOY perch in Lake Annecy are responsible for the high mortality of YOY perch during fall and first winter, as previously hypothesized for other nutrient limited lakes (Dettmers, Raffenberg, & Weis, 2003).

An alternative hypothesis is that the lower perch recruitment in Lake Annecy could be related to the occurrence of the parasitic tapeworm *T. nodulosus. Triaenophorus nodulosus* is currently recorded in almost all waters of Europe where its final host, northern pike (*Esox lucius)*, normally occurs, but its prevalence rate in YOY perch can be highly variable (Dettmers et al., 2003). In Lake Annecy, more than 80% of YOY perch are parasitized by the end of the summer, while the parasite prevalence is much lower in the other two lakes (Jacquet et al., 2015; Perga et al., 2016). *Triaenophorus nodulosus* has a three‐host life cycle where it uses copepods as the first intermediate host, a broad range of fish species (>70 spp.) as the second intermediate host (e.g., European perch, see Kuperman, 1973; Kuchta et al., 2007), and northern pike as the final host to complete its life cycle. In European perch, plerocercoids, that is, cysts in mature form of infectious stages, are concentrated in the liver and are occasionally seen in other organs such as the spleen, gonad, kidney, and musculature (Brinker & Hamers, 2007; Kuperman, 1973). The histological consequences of parasite activity in perch include lysis of the membrane of host cells and pathological symptoms such as inflammation, atrophy, necrosis, and oedema (Kuperman, 1973). Agreement on whether these symptoms have a cascading impact on the survival rate is lacking (Dieterich & Eckmann, 2000; Hoffmann, Meder, Klein, Osterkornj, & Negele, 1986). The parasitic hypothesis posits that the high prevalence of *T. nodulosus* in YOY perch in Lake Annecy affects their physiological traits and limits their physical condition at the end of their first summer and thus compromises YOY perch survival during their first winter, as compared to the other two lakes. The third and final hypothesis is that both nutritional and parasitic constraints interact to limit YOY perch survival in Lake Annecy.

## 2 MATERIALS AND METHODS

### 2.1 Study sites, sampling, and processing

**Figure 1:**
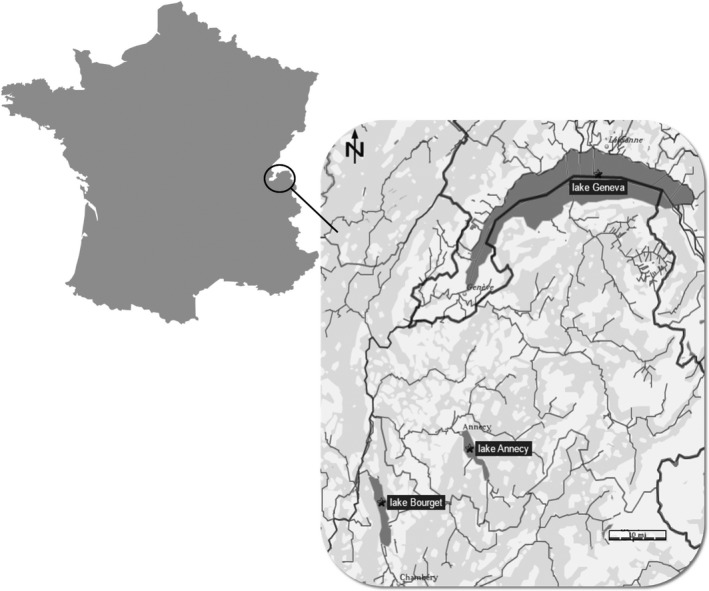
Location of study lakes

Lakes Geneva, Bourget, and Annecy are large, deep, clearwater, and monomictic lakes lying to the north‐western border of the French Alps (Figure 1, see Table 1 for characteristics). Fish communities are similar and typical of subalpine lakes with native or early introduced populations of whitefish (*Coregonus lavaretus*), European perch (*Perca fluviatilis)*, roach (*Rutilus rutilus*), and pike (*Esox lucius*). The three lakes are monitored fortnightly to monthly by OLA, the Observatory of LAkes (http://www6.inra.fr/soere-ola, see Table 1).

**Table 1:** Characteristics of the three peri‐alpine lakes studied (data for year 2015 from http://si-ola.inra.fr ©SOERE OLA‐IS, AnaEE‐France, INRA Thonon‐les‐Bains, CISALB, CIPEL, SILA, developed by Eco‐Informatics ORE INRA Team)

	Trophic status	Altitude (m)	Total area (km^2^)	Maximum depth (m)	Mean depth (m)	Phosphorus concentration (μg/L)	Zooplankton density (ind/m^3^)	Zooplankton groups
Lake Annecy	Oligotrophic	447	26	65	42	4	6,500	Cyclopoids: 50%–70% Cladocerans: 25% Calanoids: 15%
Lake Bourget	Mesotrophic	232	44	146	85	17	5,700	Calanoids: 40%–50% Cladocerans: 20%–30% Cyclopoids: 20%–30%
Lake Geneva	Mesotrophic	372	580	310	152	19	8,000	

Fish were caught in early fall 2015 during fish monitoring surveys using gillnets in multiple sites all around lakes Annecy and Bourget, following the European standard gillnet protocol (CEN 2005). Lake Geneva was sampled at only one site. Fish from all sites were identified to species, weighed to the nearest gram (wet weight), and measured (total length) to the nearest millimetre immediately after sampling. Individuals were stored at −20°C until further analysis. Lakes Annecy and Bourget captures were randomly subsampled, but all Lake Geneva catches were used.

After thawing, fish livers were excised and dissected, and the number and the wet weight of cysts were recorded. Guts were removed, and fish were decapitated and scaled. Fish bodies were then freeze‐dried and ground with a mortar and pestle for fatty acid and stable isotope analyses. A total of 424, 210, and 109 individual YOY perch were analysed for lakes Annecy, Bourget, and Geneva, respectively. Thereafter, fish were classified within the three different infection levels as defined by Brinker and Hamers (2007; ca. none, moderate ≤3 cysts, high >3 cysts in the liver), and 10–50 individuals were randomly selected within each infection levels for stable isotope and 4–19 individuals for fatty acids analyses (Supplementary Information Table S1).

### 2.2 Parasitic burden

Infection level was categorized as defined above. The parasitic index (PI, Masson, Vanacker, Fox, & Beisel, 2015) was computed as PI = (*W*_p_/*W*_f_) × 100, *W*_p_ being the total weight of cysts and *W*_f_ the total weight of the individual fish, in g. A potential physiological effect of the parasite on YOY perch was also assessed through the hepatosomatic index (HSI, Masson et al., 2015) with = ((*W*_l_−*W*_p_)/*W*_f_) × 100, *W*_l_ being the total weight of liver in g, including cysts in case of infection.

### 2.3 Stable isotope analyses

Homogenized freeze‐dried fish tissue was weighed (1 mg) into tin capsules. δ^13^C and δ^15^N were measured at the SINLAB (New Brunswick, Canada) on a Finnigan Delta Plus mass spectrometer interfaced via a Conflo II to a NC2500 Elemental. Internal laboratory standards (acetanilide, nicotinamide, and bovine liver) were run every 10 samples. The precision values of the carbon and nitrogen isotope analyses were <0.2‰ (1 *SD*). Overall, the molar C/N ratio, which had an average value of 3.4, was not significantly correlated with the fish δ^13^C values. These observations are consistent with those by Skinner, Martin, and Moore (2016), which rejected any significant bias in the lipid content of fish muscle δ^13^C below a 5% lipid content (or molar C/N < 3.5).

Variability in YOY perch δ^13^C, δ^15^N, and molar C/N was initially analysed within a single lake population. With regard to YOY perch fast growth during their first summer (Masson et al., 2001), the stable isotope composition of their body tissue was considered as an integration of that of their food sources over the last life stage (Perga & Gerdeaux, 2005). Individual YOY perch δ^13^C was used as an indicator of the intrapopulation variability in trophic habitats (littoral vs. pelagic), while δ^15^N reflected the intrapopulation variability in trophic position (zooplanktivorous vs. predatory). Due to a lack of baseline material, we did not compute any quantitative estimates for the proportion of each carbon source nor any trophic levels for individuals. Molar C/N can be considered as a surrogate for fat content in fish fillets and therefore included as an indicator for fish physiological conditions, rather than a trophic marker per se.

### 2.4 Fatty acid analyses

Briefly, the lipids were extracted using a 4:2:1 chloroform/methanol/water mixture (Parrish, 1999). Fatty acids (FA), analysed as methyl esters (FAME), were prepared by saponification of the lipid extract followed by esterification. The FAME extract was subsequently analysed by gas chromatography (GC‐2010, Shimadzu), on a Supelcowax 10 capillary column (30 m length, 0.25 mm inner diameter, and 0.25 μm film thickness), and measured by a flame ionization detector (FID). FAME were identified by comparison of their retention times with known standards (37‐component FAME mix, Supelco 47885‐U) and quantified with reference calibration curves. FA compositions are expressed in percentage of total identified FA for all sample types.

Fatty acid analyses were used as indicators of both perch trophic habitats (through the use of fatty acid trophic markers—FATM) and perch physiological conditions (through their contents in essential fatty acids, see next section). The FATM concept is based on the observation that organic sources of different origins and natures lay down certain fatty acid patterns that may be transferred conservatively to, and hence can be recognized, in consumers (Dalsgaard, John, Kattner, Müller‐Navarra, & Hagen, 2003). Among prey of YOY perch, cladocerans and cyclopoid copepods are rich in EPA (eicosapentaenoic acid; 20:5n‐3) while highly deprived in DHA (docosahexaenoic acid; 22:6n‐3) as compared to calanoid copepod species (Kainz et al., 2005; Syväranta & Rautio, 2010). Therefore, the EPA/DHA ratio in YOY perch is indicative of the (lower) share of calanoids in the diet. The ratio of bacterial FA (15:0 + 15:1 + 17:0 + 17:1) relative to the ubiquist eukaryotic 16:0 is indicative of the involvement in the detrital microbial loop within the food chain, while the terrestrial index (C24:0/C16:0) is indicative of the relative role of terrestrial carbon in supporting the secondary production (Dalsgaard et al., 2003). Hence, FA composition of YOY perch provides information on their nutritional sources and how they vary between individuals and lakes.

### 2.5 Quantification of perch physiological condition

YOY perch condition by the end of summer was first evaluated through the allometric relationship between fish weight (in g) (*W*_f_) and length (in mm) (*L*_f_): *W*_*f*_ = *a* × *L*_*f*_^*b*^ (Le Cren, 1951), where *b* is the coefficient balancing the dimensions of the equation. Individuals with negative allometry (*b* < 3) grew faster in length than in weight, suggesting they are in lower physiological conditions than those with isometry (*b* = 3) or positive allometry (*b* > 3) (Lleonart, Salat, & Torres, 2000). YOY growth in the three lakes was compared to the general equation proposed by Giannetto, Carosi, Franchi, La Porta, and Lorenzoni's (2012) model based on 64,913 specimens from 762 populations of Eurasian perch (*W*_f_ = 0.7·10^−5^ × *L*_f_^3.101^).

Furthermore, the availability of highly unsaturated fatty acids, and especially EPA and DHA, is of prime importance for larval and juvenile development and fitness (Sargent, Bell, McEvoy, Tocher, & Estevez, 1999), larval condition and juvenile behaviour, growth rate, ability to feed, and development of the brain and nervous systems (Ishizaki et al., 2001). DHA and other long‐chain fatty acids may also be determinant for antipredator performance of fish larvae, with effects on escape responses mediated by sensory systems (Fuiman & Ojanguren, 2011). Therefore, the retention and accumulation of these essential FA, which cannot be synthesized by fish, in the early larval stages are key factors in fish recruitment fluctuations (Bell & Sargent, 1996). Percentage contents in these FA were then considered as secondary indicators of fish physiological conditions, such as C/N molar ratios.

### 2.6 Data analyses

Parasite occurrence and prevalence between lakes were compared by using Chi‐squared tests. Differences in average length and weight of fish, parasitic and hepatosomatic indexes (PI, HSI), and biomarker levels were tested, between lakes and infection levels, by using ANOVA. Relationships between fish condition and parasitism were analysed both between lakes and within lakes. ANCOVA was first used to detect differences in allometric coefficients between lakes and to test whether they diverged from theoretical values for isometric growth. In the regression models, log(Weight) was the dependent variable and log(Length) the covariate, lake identity being the categorical variable. The slope of the relationships provides an estimate (expected mean ± 95% confidence interval) of the allometric coefficient which can be readily compared to the theoretical value for isometric growth (within or out of the 95% CI) and between each lake (whether confidence intervals intercross or not). Because allometric coefficients were found to vary between lakes, infection level was thereafter introduced as a nested factor within lakes, to potentially detect lake‐specific parasitic effect. Differences in FA composition (arcsin square root transformed to achieve normality) and biomarkers levels between lakes and infections were explored by using linear discriminant analysis (LDA) and multivariate ANOVA (MANOVA). All tests were performed with R 3.1.1 software (R Core Development Team, 2008) with the alpha risk set at 5%.

## 3 RESULTS

### 3.1 Parasite load of *T. nodulosus* on the population of YOY perch in the three studied lakes

Parasite prevalence was the highest in Lake Annecy, with 83% of YOY perch containing liver cysts of *T. nodulosus*. Prevalence was the lowest in Lake Bourget (61%) and intermediate in Lake Geneva (77%). The distribution (*χ* ^2^ = 62, *p* < 0.001) and average number of liver cysts per individuals (ANOVA, *F *=* *118, *p *<* *0.001) differed significantly between lakes. In Lake Annecy, individuals carried on average 1.79 cysts as compared to 1.13 and 1.34 for lakes Bourget and Geneva, respectively. PI was 0.62 for YOY perch in Lake Annecy, as compared to 0.18 and 0.28 for lakes Bourget and Geneva, respectively.

### 3.2 Relationships between life history traits and parasitism between‐ and within‐lake populations

**Figure 2:**
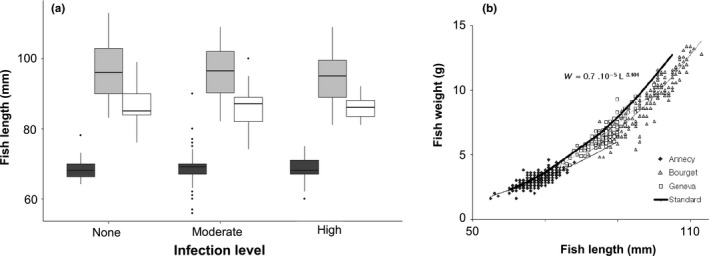
Differences in fish growth parameters. (a) Boxplots (median, quartiles and outliers) presenting the length distribution of YOY perch (*Perca fluviatilis*) between lakes (dark grey for L. Annecy, light grey for L. Bourget, and white for L. Geneva) and infection levels of *Triaenophorus nodulosus* (from none to high). (b) Allometric length–weight relationships for YOY perch (*Perca fluviatilis*) from lakes Annecy, Bourget, and Geneva, as compared to the equation of Giannetto et al.'s (2012) model (*W*_f_ = 0.7·10^−5^×*L*_f_^3.101^)

Average YOY perch length (Figure 2a) and weight were significantly different among lakes (one‐way ANOVA, *F *=* *2440, *p *<* *0.001, and *F *=* *1550, *p *<* *0.001, for length and weight, respectively). YOY perch from Lake Annecy were approximately 30% shorter and 55% lighter than those from Lake Bourget at similar infection levels (Figure 2). Neither YOY length nor weight varied significantly according to infection levels within lakes (two‐way ANOVA for length, infection levels, *F *=* *0.025, *p *=* *0.98).

Allometric relationships also differed significantly between lakes (Table 2, Figure 2b). Estimated values for the allometric coefficient pointed to an isometric growth in both lakes Geneva and Bourget, while in Lake Annecy YOY perch clearly grow with a negative allometry (Figure 2b). Within each lake though, *b* values did not vary according to the infection level, that is, as for length and weight. Therefore, YOY growth was not impacted by *T. nodulosus* infection level.

**Table 2:** Allometric relationships of YOY perch (*Perca fluviatilis*) body condition (length/weight) by linear models tested with ANCOVAs between lakes and within lakes between infection levels (from none to high)

ANCOVA (model)		*F*	*p*	Effect	Estimated value of the allometric coefficient (95% CI)
Lake effect		29.6	4·10^−13^	Annecy	2.44 (2.26–2.61)
				Bourget	3.04 (2.88–3.19)
				Geneva	2.91 (2.69–3.12)
Infection level within lakes	Annecy	1.24	0.30		
	Bourget	0.98	0.38		
	Geneva	0.39	0.68		

**Figure 3:**
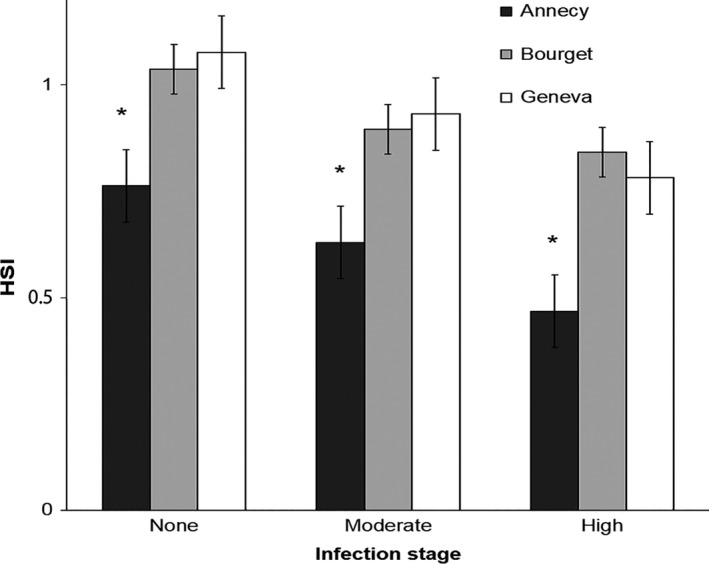
Average (±*SE*) values of the hepatosomatic index (HSI) for YOY perch (*Perca fluviatilis*) between lakes and infection levels of *Triaenophorus nodulosus*. (* indicates significant differences at the 5% alpha risk between Lake Annecy and the two other lakes Geneva and Bourget)

The HSI varied significantly between lakes (Table 3) and showed higher values for YOY perch in lakes Bourget and Geneva as compared to Lake Annecy. Within each single lake, HSI decreased significantly with the infection level (Table 3, Figure 3). The strongest effect was observed for the YOY population of Lake Annecy, for which HSI was 35% lower for the highest infection level than for no infection.

**Table 3:** Results from ANOVAs testing whether indicators of YOY perch (*Perca fluviatilis*) (hepatosomatic index, stable isotopes, and trophic biomarkers as terrestrial index and ratio EPA/DHA) varied between lakes and within lakes for each category of infection level (from none to high)

Response (model)	Lake effect		Infection levels within lakes		
	*F*	*p*	Lake	*F*	*p*
HSI	17.40	3·10^−7^	Annecy	63.9	1·10^−14^ a
			Bourget	9.0	0.003^a^
			Geneva	11.4	0.001^a^
δ^13^C	462.7	<2·10^−16^	Annecy	0.64	0.53
			Bourget	1.21	0.31
			Geneva	3.66	0.04^a^
δ^15^N	1,070	<2·10^−16^	Annecy	0.34	0.71
			Bourget	1.94	0.16
			Geneva	0.24	0.79
C:N	19.84	6·10^−8^	Annecy	3.87	0.03^a^
			Bourget	2.13	0.14
			Geneva	0.32	0.73
24:0/16:0 terrestrial	4.68	0.01	Annecy	2.17	0.13
			Bourget	4.80	0.02^a^
			Geneva	2.98	0.07
EPA/DHA	12.63	1·10^−5^	Annecy	1.18	0.32
			Bourget	4.36	0.02^a^
			Geneva	3.37	0.05^a^

a Significant differences at the minimum 5% alpha risk level.

### 3.3 Isotopic, elementary, and fatty acid biomarkers

**Figure 4:**
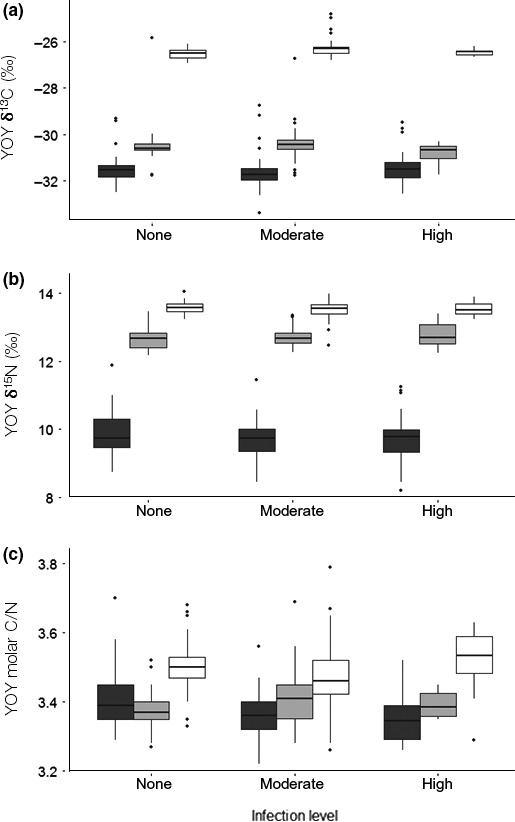
Boxplots (median, quartiles, and outliers) presenting (a) δ^13^C, (b) δ^15^N, and (c) molar C/N of YOY perch (*Perca fluviatilis*) for lakes (dark grey for L. Annecy, light grey for L. Bourget, and white for L. Geneva) and infection levels of *Triaenophorus nodulosus* (from none to high)

**Figure 5:**
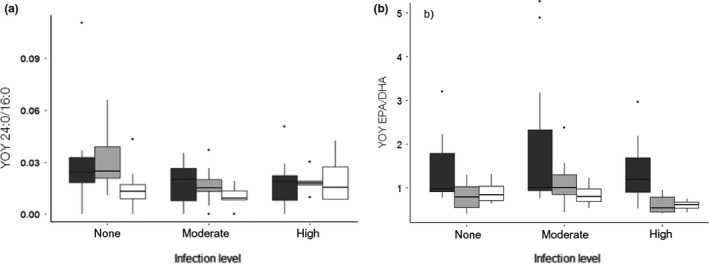
Boxplots (median, quartiles, and outliers) presenting (a) 24:0/16:0 ratio, that is, terrestrial index and (b) EPA/DHA ratio of YOY perch (*Perca fluviatilis*) for lakes (dark grey for L. Annecy, light grey for L. Bourget, and white for L. Geneva) and infection levels of *Triaenophorus nodulosus* (from none to high)

Fish δ^13^C, δ^15^N, and C/N varied significantly between lakes (Table 3, Figure 4), suggestive of contrasting trophic niches. δ^13^C and δ^15^N values were the lowest in Lake Annecy, intermediate in Lake Bourget, and the highest in Lake Geneva. Perch δ^13^C and δ^15^N were not correlated with fish length and did not vary significantly among infection levels within lakes. Fatty acid composition of YOY varied significantly among lakes (LDA, *Wilks’ lambda* = 0.05, *F*(2,48) =* *110, *p *<* *10^−15^) but not infection levels (LDA, *Wilks’ lambda = *0.59, *F*(2,48) =* *1.03, *p = *0.44). The differences between lakes were the most striking for percentages of some mono‐ and polyunsaturated FA (PUFA) (Supplementary Information Table S2), EPA/DHA, and the terrestrial index (Table 3). C/N ratios (Figure 4c) and relative abundances in PUFA (Supplementary Information Table S2) were the lowest in Lake Annecy, along with the highest terrestrial index 24:0/16:0 (Figure 5a) and EPA/DHA (Figure 5b). At the other end of the scale, YOY perch of Lake Geneva exhibited high C/N ratios (Figure 4c), high proportions of PUFA (Supplementary Information Table S2), and comparatively the lowest values for the EPA/DHA and terrestrial index (Figure 5). In Lake Annecy, the composition in lipid biomarkers was not dependent on the infection levels but C/N tended to be lower for the moderate and high infection levels. In lakes Bourget and Geneva, the physiological EPA/DHA ratio (Table 3, Figure 5) displayed values that were significantly lower for the higher infection levels.

## 4 DISCUSSION

The paucity of *in situ* examples of the dynamic consequences of parasites on their host reflects the difficulties inherent in teasing out the role of parasites in complex ecological systems with many potentially confounding factors (Albon et al., 2002; Britton, Pegg & Williams, 2011; Iwanowicz, 2011). Indeed, a small but growing field of evidence exists to support the theory that macro‐parasites play a role in regulating host populations (Albon et al., 2002).

If work on the ecological impacts of parasites in the field is necessarily correlative (Poulin, 2007), identification of the mechanisms that lead to differences detected between infected and noninfected hosts is difficult due to preexisting differences that may have conditioned the success of infection (Bize, Jeanneret, Klopfenstein, & Roulin, 2007), or the physiological consequences of the infection (Anderson & May, 1978). The energetic costs of parasitism can be direct, by diverting host energy allocation, or indirect, due to behavioural changes (Loot, Brosse, Lek, & Guégan, 2001) or modifications in host metabolic rates and energy needs (Booth, Clayton, & Block, 1993). The theory of the parasitic cost itself can be contested even at the individual scale because, counter‐intuitively, growth of infected hosts can exceed that of noninfected individuals (Arnott, Barber, & Huntingford, 2000). As the impacts of parasitism may depend, even at the individual scale, on the hosts nutritional state (Washburn et al., 1991), evaluation of the actual role of parasitism to control the survival of individuals and then the dynamics of a host population remains very difficult.

### 4.1 A primary nutritional control on YOY perch growth and fat storage

The physiological state and life history traits of the YOY perch populations clearly varied between the three study lakes, while differences of these same parameters between infection levels within lakes were less significant. Therefore, the major controlling factor of perch growth and physiology in our case study was acting at the lake scale (rather than within a given lake).

At the end of their first summer, YOY perch in Lake Annecy were smaller and lighter than in the other two lakes, and they also grew anisometrically (i.e., they had a mass deficit before their first winter). The liver is the dominant organ for lipid deposition in juvenile fish (Sargent, McEvoy, et al., 1999); hence, HSI is directly depending on the lipid content of the fish diet. In Lake Annecy, lower C/N ratios of the total body mass for YOY perch and lower values of HSI suggest that lower growth was associated with a deficit in fat storage at the end of the summer. Winter mortality of age 0+ perch is size selective, such that larger individuals (a) are expected to more easily withstand winter food limitation and fat depletion (Post & Evans, 1989), (b) are more likely to switch to piscivory and cannibalism before winter, a more energetically rewarding food source (Keast & Eadie, 1985), and (c) should escape predation and cannibalism (Persson, Bystrom, Wahlstrom, & Westman, 2004). However, Huss, Byström, Strand, Eriksson, and Persson (2008) demonstrated that winter survival for age 0+ yellow perch (*Perca flavescens*) in Canadian lakes was functionally more dependent on fat storage than on individual size. Abiotic factors such as water temperature and/or wind can influence perch growth during their first year (Mélard, Kestemont, & Grignard, 1996), but Lake Annecy, being the warmest of these three lakes and also the most sheltered from winds (Perga et al., 2015), would present the best abiotic conditions for fish growth. Therefore, water temperature and wind are unlikely factors limiting perch growth in Lake Annecy, and the amount of fat storage is likely driven more by the feeding history of juveniles prior to the winter (Heermann, Eriksson, Magnhagen, & Borcherding, 2009).

YOY perch feed on zooplankton and preferentially on copepods (calanoids and cyclopoids) (Guma'a, 1978; Masson et al., 2001). Calanoids are more nutritionally rewarding than cyclopoids and cladocerans (Smyntek, Teece, Schulz, & Storch, 2008), being at the same time fattier, but also richer in docosahexaenoic acid (DHA), a long‐chain polyunsaturated fatty acid that is essential for juvenile fish growth and development (Sargent, Bell, et al., 1999; Sargent, McEvoy, et al., 1999). Total annual zooplankton density in Lake Annecy is 6,500 ind./m^3^ and intermediate between that of Lake Bourget (5,700 ind./m^3^ in 2015) and Lake Geneva (approximately 8,000 ind./m^3^, in 2015, Table 1), in spite of its lower phosphate concentrations. Therefore, zooplankton quality (Arts & Sprules, 1989) instead of quantity might be the bottleneck for perch growth in Lake Annecy. In lakes Geneva and Bourget, *Eudiaptomus gracilis,* the only calanoid species*,* makes up 40%–50% of microcrustacean community, while cladocerans (*Daphnia longispina* sp. and *Bosmina* sp.) and cyclopoid copepods (*Cyclops* sp.) contribute to 20%–30% each. In contrast, in Lake Annecy, the microcrustacean community is largely dominated by *Cyclops* sp. (50%–70% of the community), while cladocerans represent 25%. Calanoids, represented by the only species *Mixodiaptomus laciniatus*, rarely makes up >15% of the microcrustacean community (data from http://si-ola.inra.fr). Dominance by omnivorous and opportunistic cyclopoid copepods is usually expected in oligotrophic systems for which pico‐ and nano‐plankters make up an important share of primary production (Sommer, Stibor, Katechakis, Sommer, & Hansen, 2002), or for which the microbial loop, that is, the trophic pathway where dissolved organic carbon (DOC) is returned to higher trophic levels via its incorporation into bacterial biomass, plays an important role in remobilizing detritus in these systems with low autotrophic productions (Wickham, 1995). Both conditions are met in Lake Annecy. Pico‐ and nano‐plankters represent typically 50% of the phytoplankton biomass (Perga et al., 2016), while bacterial grazing by mixotrophic flagellates makes the brown chain, that is, food web based on detritus, highly efficient (Domaizon, Viboud, & Fontvieille, 2003). Cyclopoid copepods are the most efficient zooplankters to exploit both these pico‐autotrophic and heterotrophic food sources in a context of nutrient limitation. Evidence of terrestrial supplementation of the secondary production in the oligotrophic Lake Annecy has also been provided in a previous study, in which the high content in the terrestrial indicator 24:0/16:0 for *Cyclops* and juvenile whitefish in Lake Annecy confirmed the role of *Cyclops* as the crucial trophic node connecting the brown chain and fish (Perga, Bec, & Anneville, 2009). The contribution of terrestrial organic matter also appeared for YOY perch in Lake Annecy and is consistent with the control of nutrient limitation on zooplankton structure, which is further transmitted up to fish growth. In contrast, the calanoid *Eudiaptomus gracilis* is highly ubiquitous and its presence cannot be straightforwardly related to lake trophic status (Riccardi & Rossetti, 2007). Instead, *E. gracilis* is thought to be favoured in aquatic systems in which blooms of large or toxic algae occur (Sommer et al., 2002), such as in lakes Geneva and Bourget where blooms can occur episodically (Jacquet et al., 2005; Tapolczai et al., 2015).

If the underlying processes by which nutrients control the microcrustacean community structure in these lakes cannot yet be fully explained, they undeniably have consequences in terms of the quality of the food available for YOY perch. In fact, YOY perch were not only fattier in the two lakes in which calanoids dominated zooplankton, but they also exhibited higher levels of DHA and lower EPA/DHA ratios. The results confirm that perch growth and probability to survive their first winter in Lake Annecy were probably limited by food quality in this lake, as compared to lakes Geneva and Bourget.

### 4.2 Parasitism impact on perch growth and fat storage

The fish tapeworm *T. nodulosus* is the most pathological species of the *Triaenophorus* genus (Brinker & Hamers, 2007). Controversial data exist on the effects of *T. nodulosus* plerocercoids on European perch growth and condition, but the increase in prevalence and abundance has been considered as one reason for diminishing perch stocks in Lake Constance (Dieterich & Eckmann, 2000). Indeed, infected individuals in Lake Constance had a lower growth than uninfected individuals, but only for individuals older than 2 years. According to Brinker and Hamers (2007) and Dezfuli, Giari, Lorenzoni, and Noga (2014), pathological effects occur when infection level exceeds three cysts/individual. In Lake Annecy, highly infected YOY perch represent 25% of the population versus 11%–12% for the other two lakes.

For the present study, despite the high infection levels, we did not find evidence for any significant difference in fish growth within lakes that could be attributed to the parasite effect. Even individuals with a high parasitic load were not statistically smaller or lighter than those that were not infected, in any of the lakes. HSI was the only parameter which showed a consistent effect of infection levels along with a lake effect, meaning that part of the fat deficit observed by the end of summer for Lake Annecy could be partly due to a deleterious effect of *T. nodulosus* on liver size and function. However, liver tissue lost during excising of the parasite can affect results; the more parasites removed the greater the possibility removing liver tissue too. Yet, *Raphidascaris acus*, which, like *T. nodulosus*, encysts in the liver of yellow perch (*Perca flavescens*), significantly diminishes fat storage of the host (Johnson & Dick, 2001). However, in Lake Annecy, final effect on body fat (C/N) was only significant for the highly infested YOY perch, while moderate to highly infested perch in lakes Geneva and Bourget exhibited higher DHA levels (and subsequent lower EPA/DHA ratios), suggesting that for the other two lakes, the availability of higher‐quality food might compensate for the parasitic effect on the quantity and quality of lipid reserves. Therefore, *T. nodulosus* effect on fat storage, which, as discussed above, plays a crucial role in winter survival, could be a secondary factor contributing to the low perch recruitment in lakes for which food quality is already limiting.

### 4.3 Nutritional and parasitic control on perch feeding behaviour

YOY perch exhibit an ontogenic change in feeding behaviour, shifting from planktivory to benthivory and/or piscivory. The timing and succession of these shifts depend on the lake feeding resource levels (Hjelm, Svanback, Bystrom, Persson, & Wahlstrom, 2001). Beyond direct impacts, consequences of parasitism on host, particularly with trophically transmitted parasites, could also trigger changes in feeding behaviour and habitat use of the intermediate hosts to increase susceptibility to predation by final hosts (Loot et al., 2001). For the YOY perch—*T. nodulosus* model, the most advantageous behavioural modification for the parasite would be an earlier shift of YOY perch to littoral habitats to increase the probability of encountering its final host, that is, pike. The ultimate effects of such indirect consequences of the parasite on host growth could mimic those of their direct effects as a shift in habitat utilization may lead to a change in food availability and quality. Changes in perch feeding behaviour, whether due to nutritional or to parasitic control, should in theory be detected from analysis of the stable isotope composition of body tissue.

There were significant differences in YOY perch δ^13^C between lakes, but these are actually due to already well‐documented isotopic baseline effects as planktonic δ^13^C values naturally increase with lake size and trophic status for these peri‐alpine lakes (Perga & Gerdeaux, 2004). YOY perch δ^13^C varied only marginally within lakes with parasitic infection level. Because fish δ^13^C can vary according to the lipid content of fish body (Skinner et al., 2016), variation in δ^13^C could also be the consequence of highly infested fish also being slightly less fat. Therefore, we considered that these small δ^13^C differences are not robust enough to confidently pinpoint a parasite effect on perch shift to littoral habitat, tied to increasing δ^13^C.

The annual mean and range of seasonal variability in δ^15^N for zooplankton are comparable between lakes Annecy and Geneva, while we have no data for Lake Bourget. YOY perch δ^15^N in lakes Geneva and Bourget were about 4‰ higher than in Lake Annecy at the end of summer, that is, quite consistent for what is expected for a shift from zooplanktivory to piscivory (Persson & Greenberg, 1990). Within lakes, individual δ^15^N was not significantly correlated with fish size, as a result of low intralake variability in both parameters. Because the timing of perch ontogenic shift depends more on morphometric changes and lake resource levels than on absolute size alone, it is likely that the high food quality in lakes Bourget and Geneva allows for the whole perch cohort to shift to piscivory before the end of summer. In contrast, in Lake Annecy, the perch cohort did not achieve the growth threshold for piscivory by the end of summer, yet another factor making them more vulnerable to winter kills (Hjelm, Persson, & Christensen, 2000). δ^15^N of perch did not vary significantly between infection levels within lakes, excluding a potential parasitic driven change in the timing of the ontogenic shift.

### 4.4 Co‐occurrence of low food quality and parasite loads

Overall, we found a low impact of parasitism on the physiological condition and trophic level of YOY perch at the end of their first summer in lakes Geneva, Annecy, and Bourget and speculated by extension, on their winter survival and recruitment. We suspect, however, that parasitism by *T. nodulosus* had a significant effect on liver lipogenic activity at higher levels of infections that contributes to the deficit in fat storage for YOY perch that were already in a nutritionally limited environment. Hence, we consider parasitic control of the host population is of secondary importance, conditioned by the primary nutritional constraint. Therefore, the high mortality of perch in late fall and winter in Lake Annecy is not caused by the higher parasite prevalence, but instead, both phenomena are most likely driven by the stoichiometric constrain in this lake. The very low phosphorous concentrations favour zooplankters with low P requirements, that is, cyclopoids, over the P‐demanding cladocerans and calanoids (Andersen & Hessen, 1991). Cyclopoid copepods become dominant in peri‐alpine lakes with low nutrient concentrations, as Lake Annecy, and given the role of these cyclopoid copepods as intermediate hosts in the life cycle of *T. nodulosus,* the probability for YOY perch to encounter an infested prey is higher (Brinker & Hamers, 2007). Our results suggest an indirect link between environmental factors (lake phosphorus concentrations) and the prevalence of an endoparasite, through the modulation of the parasite–host encounter rates in the system *Perca fluviatilis–Triaenophorus nodulosus*, shedding light on the need to consider abiotic factors but also the pathways by which they can act on the host–parasite relationships in nature.

## CONFLICT OF INTEREST

None declared.

## AUTHORS' CONTRIBUTION

MEP and JG conceived the study; AF analysed the samples and performed the statistical analyses; AF, MEP, and JG drafted the manuscript.

## DATA ACCESSIBILITY

Fish metrics, parasitic data, stable isotope, and fatty acid contents are available from the Dryad Digital Repository: https://doi.org/10.5061/dryad.2kf5b8k.

Lake Data: publicly available on http://si-ola.inra.fr.
