# Supplementary material for: Parasitic versus nutritional regulation of natural fish populations
Source: Ecol Evol. 2018 Aug 5;8(17):8713–25. doi: 10.1002/ece3.4391 (PMC6157692; doi:10.1002/ece3.4391)
Supplement: Supplementary file 1 [file ECE3-8-8713-s001.docx]

Supplementary material

**Parasitic versus nutritional regulation of natural fish populations.**

Amélie Frantz^1,2^, Marie-Elodie Perga*^1,3^, Jean Guillard^1^**.**

^1^UMR 042 CARRTEL, INRA - University Savoie Mont Blanc, 75 av. de Corzent, Thonon les Bains F-74203, France

^2^University of Bordeaux, EPOC, UMR 5805, LPTC Research Group, Talence F-33400, France

^3^Institute of Earth Surface Dynamics, University of Lausanne, Lausanne, CH-1015, Switzerland

*** :** corresponding author: [marie-elodie.perga@unil.ch](mailto:marie-elodie.perga@unil.ch)

| **Lake** | | **Annecy** | | | **Bourget** | | | **Geneva** | | |
| --- | --- | --- | --- | --- | --- | --- | --- | --- | --- | --- |
| **Number of liver cysts** | | **0** | **<3** | **≥3** | **0** | **<3** | **≥3** | **0** | **<3** | **≥3** |
| Number of analysed individuals | Parasitic load and Morphometry | 74 | 273 | 120 | 81 | 106 | 23 | 25 | 69 | 15 |
|  | Stable isotope analyses | 31 | 47 | 30 | 29 | 50 | 10 | 25 | 50 | 10 |
|  | Fatty acid analyses | 11 | 17 | 16 | 10 | 18 | 5 | 10 | 19 | 4 |

Table SI1. Distribution of the *Triaenophorus nodulosus* cysts in the liver of YOY perch (*Perca fluviatilis*) in catches from the three lakes. Distribution of individuals selected for stable isotope and fatty acid analyses within the range of infection levels for each single lake.

|  |  | **Lake effect** | | **Infection effect** | | **Infection within Lake effect** | |
| --- | --- | --- | --- | --- | --- | --- | --- |
|  |  | F | p-value | F | p-value | F | p-value |
| Saturated FA | **14:0** | 0.81 | 0.45 | 0.35 | 0.56 | 0.74 | 0.48 |
|  | **15:0** | 3.21 | 0.05 | 0.26 | 0.61 | 0.28 | 0.75 |
|  | **16:0** | 0.81 | 0.77 | 0.09 | 0.77 | 0.75 | 0.48 |
|  | **17:0** | 4.48 | 0.02* | 0.17 | 0.68 | 0.20 | 0.82 |
|  | **18:0** | 5.42 | 0.007* | 0.04 | 0.84 | 1.21 | 0.31 |
|  | **20:0** | 3.34 | 0.04* | 0.11 | 0.74 | 0.61 | 0.55 |
|  | **21:0** | 1.48 | 0.23 | 1.31 | 0.26 | 1.08 | 0.35 |
|  | **22:0** | 1.52 | 0.23 | 2.26 | 0.14 | 0.95 | 0.39 |
|  | **24:0** | 3.57 | 0.03* | 6.99 | 0.01* | 0.51 | 0.60 |
| Monounsaturated FA | **14:1** | 1.82 | 0.17 | 1.38 | 0.25 | 1.39 | 0.26 |
|  | **15:1** | 0.96 | 0.39 | 1.03 | 0.32 | 0.88 | 0.42 |
|  | **16:1** | 1.73 | 0.18 | 0.05 | 0.83 | 0.38 | 0.68 |
|  | **18:1(n-9)** | 1.73 | 0.19 | 0.62 | 0.44 | 0.67 | 0.51 |
|  | **20:1(n-9)** | 8.26 | 0.0007* | 0.03 | 0.86 | 0.41 | 0.67 |
|  | **24:1(n-9)** | 12.19 | 3.92e^-5*^ | 0.37 | 0.54 | 0.40 | 0.67 |
| Polyunsaturated FA | **18:3(n-6)** | 5.44 | 0.007* | 3.38 | 0.07 | 1.32 | 0.27 |
|  | **18:3(n-3)** | 6.40 | 0.003* | 0.24 | 0.63 | 0.49 | 0.61 |
|  | **20:3(n-3)** | 9.15 | 0.0004* | 1.99 | 0.16 | 1.14 | 0.33 |
|  | **20:4(n-6)** | 15.19 | 5.12e^-6*^ | 0.36 | 0.55 | 0.83 | 0.44 |
|  | **20:5(n-3)** | 0.72 | 0.49 | 1.56 | 0.22 | 0.82 | 0.45 |
|  | **22:6(n-3)** | 2.17 | 0.12 | 0.01 | 0.90 | 1.20 | 0.31 |

Table SI2. Summary of the results of MANOVA Fatty Acids composition between lakes, and within lakes for different infection levels.
